# Pancreatic Progenitor Commitment Is Marked by an Increase in Ink4a/Arf Expression

**DOI:** 10.3390/biom11081124

**Published:** 2021-07-30

**Authors:** Elena Montano, Alessandra Pollice, Valeria Lucci, Geppino Falco, Ornella Affinito, Girolama La Mantia, Maria Vivo, Tiziana Angrisano

**Affiliations:** 1 Department of Biology, University of Naples “Federico II”, 80147 Naples, Italy; elenamontano26@gmail.com (E.M.); apollice@unina.it (A.P.); valeria.lucci@unina.it (V.L.); geppino.falco@unina.it (G.F.); lamantia@unina.it (G.L.M.); 2 Department of Nuclear Medicine, IRCCS—Referral Cancer Center of Basilicata (CROB), 85028 Rionero in Vulture, Italy; 3 Biogem Scarl, Istituto di Ricerche Genetiche “Gaetano Salvatore”, 83031 Ariano Irpino, Italy; 4 IRCCS SDN, 80143 Naples, Italy; ornella.affinito@gmail.com; 5 Department of Chemistry and Biology, University of Salerno, 84084 Fisciano, Italy

**Keywords:** *Cdkn2a*, embryonic stem cells, Pancreatic Progenitor Cells, ARF, INK4a

## Abstract

The identification of the molecular mechanisms controlling early cell fate decisions in mammals is of paramount importance as the ability to determine specific lineage differentiation represents a significant opportunity for new therapies. Pancreatic Progenitor Cells (PPCs) constitute a regenerative reserve essential for the maintenance and regeneration of the pancreas. Besides, PPCs represent an excellent model for understanding pathological pancreatic cellular remodeling. Given the lack of valid markers of early endoderm, the identification of new ones is of fundamental importance. Both products of the *Ink4a*/*Arf* locus, in addition to being critical cell-cycle regulators, appear to be involved in several disease pathologies. Moreover, the locus’ expression is epigenetically regulated in ES reprogramming processes, thus constituting the ideal candidates to modulate PPCs homeostasis. In this study, starting from mouse embryonic stem cells (mESCs), we analyzed the early stages of pancreatic commitment. By inducing mESCs commitment to the pancreatic lineage, we observed that both products of the *Cdkn2a* locus, *Ink4a* and *Arf*, mark a naïve pancreatic cellular state that resembled PPC-like specification. Treatment with epi-drugs suggests a role for chromatin remodeling in the *CDKN2a* (Cycline Dependent Kinase Inhibitor 2A) locus regulation in line with previous observations in other cellular systems. Our data considerably improve the comprehension of pancreatic cellular ontogeny, which could be critical for implementing pluripotent stem cells programming and reprogramming toward pancreatic lineage commitment.

## 1. Introduction

Alterations in PPCs (Pancreatic Progenitor Cells) homeostasis are fundamental to physiological processes, such as organ development and pathological events. In particular, PPCs are essential for the maintenance and regeneration of organs and tissues that exhibit high rates of cell turnover or regenerative reserve. Interestingly, events associated with an early epithelial-to-mesenchymal transition (EMT) phenotype closely resemble PPCs cellular transition that occurs during normal pancreas embryogenesis [1,2]. Experiments on genetically engineered mice revealed that depletion of progenitor cells leads to acute or delayed failure in tissue homeostasis, limited regenerative potential, and physiological aging [3]. Besides physiological aging, senescence is a growth-arrest mechanism triggered by different insults to protect cells from hyper-proliferative signals and various forms of stress, representing an important barrier against tumor formation [3].

The *CDKN2A* locus encodes two tumor suppressors, p16INK4a (INK4a) and p14ARF (ARF), and is among the most frequently mutated in human cancers [4]. Inactivation of Arf and Ink4a in mice induces tumors with complete penetrance, while in several human cancers, both epigenetic silencing and mutational inactivation of these genes have been described [5]. Interestingly, the locus is found mutated or epigenetically silenced in 30–70% of PanINs (pancreatic intraepithelial neoplasms), a percentage that becomes as high as 95% in full-blown tumors [6]. Asides from being involved in cancer, both INK4a and ARF are involved in differentiation, apoptosis, and senescence in a number of cellular contexts [7,8]. Interestingly, both INK4a and ARF are expressed during pancreas organogenesis and are tightly correlated with PPCs’ quiescence [9]. Several lines of evidence suggested that increased transcription of INK4A and ARF provokes senescence in various cell types with a causative role in age-associated degenerative diseases. Hence, the comprehension of the mechanisms regulating the expression of these genes has major implications for both cancer and degenerative disorders. In beta cells, it is known that the lack of INK4a expression leads to the overexpression of CDK4 and consequently to an increase in cell proliferation, rising insulin secretion, and causes pancreatic hyperplasia [9]. Germline *CDKN2A* mutations have also been occasionally found in pancreatic cancer patients and families without familial melanoma, in which the locus involvement has been described in several systems [10,11,12]. Variants of *CDKN2A/B* are involved in susceptibility to type 2 diabetes highlighting its possible role in Beta-cell function and regeneration [13]. Besides their function as a barrier to tumor progression, a number of studies demonstrated that both proteins are implicated in developmental processes [7].

In this study, we followed the *Ink4a*/*Arf* locus expression during pancreatic commitment in mouse ESC. We provide data showing that upon mESCs differentiation in vitro, *Ink4a*/*Arf* expression sharply increases both at a transcriptional and protein level. In particular, both ARF and INK4a are expressed during the early stages of murine endoderm specification in a subset of cells marked by the expression of the *Nepn* (*Nephrocan*) gene at E7.5–11.5 of mouse development. Furthermore, mESCs treatment with 5-aza-2-deoxyazacytidine (AZA) and trichostatin (TSA), in addition to allowing the expression of PPC markers, also induce an increase in *Ink4a*/*Arf* gene expression, thus suggesting that an epigenetic-dependent mechanism controls *Cdkn2a* locus remodeling activated during PPCs differentiation.

## 2. Materials and Methods

### 2.1. ESCs Culture and Differentiation

mESCs were cultured on 0.1% gelatine in DMEM high glucose (Gibco, Thermo Fisher Scientific, Inc., Waltham, MA, USA) supplemented with 15% fetal bovine serum (Gibco), 0.1 mM 2(β)-Mercaptoethanol (Sigma, St. Louis, MO, USA), 1 mM NEAA (non-essential amino acid) (Gibco), 2 mM L-glutamine (Gibco), and 1000 units/mL leukemia inhibitory factor (Lif) and grown at 37 °C with 5% CO_2_ in a humidified incubator. The ESCs were differentiated as described in [14,15]. Briefly, ESCs were cultured with activin A (30 ng/mL) for 4 days to obtain the definitive endoderm (DE) cells and for another 4 days in the presence of both retinoic acid (RA) (5 μM) and Fibroblast growth factor 10 (FGF10) (10 ng/mL) to obtain the Posterior Foregut Endoderm (PFE) and enrichment of PPCs. AZA (ICN Biomedical Inc., Costa Mesa, CA, United States) treatments were performed at 25 μM final concentration, while TSA (Sigma Aldrich) was used at 100 nM. The experiments in sorted cells were conducted on Nepn ESCs clone EPD0686_5_C01 obtained from Komp Repository and E14Tg2a.4 [16]. Cell suspension preparation and FACS (Fluorescence Activated Cell Sorting) were performed following published methods [17].

### 2.2. qPCR Analysis

Total RNA was isolated from cells using Trizol (Sigma) according to the manufacturer’s protocol and quantified by measuring absorption at 260 and 280 nm by Nanodrop. Purity was evaluated by gel electrophoresis and A260/A280 ratio calculation. One μg of total RNA was reverse transcribed into cDNA using iScript cDNA Synthesis Kit (Bio-Rad Laboratories, Hercules, CA, USA), according to manufacturer’s protocol. Two μL of diluted cDNA preparation (equivalent to 20 ng of starting RNA) was used as a template for the qPCR analysis.

qPCR was performed in duplicate with the SYBR Green PCR Master Mix using a 7500 Real-Time PCR System machine (Applied Biosystems, Waltham, MA, USA). Quantitative relative expression was calculated according to the 2-DDCt method (Delta Ct method), normalizing to Gapdh. All the genes analyzed and primer sequences used in the reactions are listed in Table 1.

### 2.3. Western Blot Analysis

Total proteins were extracted using 4× Laemmli Sample Buffer with the addition of 1 mM DTT (Dithiothreitol). Subcellular fractionation was carried out as described in [18]. Protein concentration was determined using the Bio-rad protein assay, and after denaturation at 90 °C for 10 min, equal amounts of proteins were electrophoretically separated through 13% SDS polyacrylamide gels transferred on polyvinylidene difluoride (PVDF) membranes. The membranes were blocked with 5% non-fat milk and probed with primary antibodies overnight at 4 °C with a gentle shaker, followed by incubation with HRP-conjugated secondary antibodies for 2 h at room temperature (RT). Proteins were detected by the ECL system (BioRad), and images were taken with ChemiDoc XRS System (Bio-Rad Laboratories) and quantified with densitometric analysis by Image J Software. Total protein extracts were normalized to Gapdh, while to verify the purity of the subcellular fractionation, Parp1 and Gapdh were used respectively as nuclear and cytoplasmic markers. The antibodies rabbit anti-p19Arf (Cayman Chemical, Ann Arbor, MI, USA), rabbit anti-p16Ink4a (Sigma Aldrich, Munich, Germany), anti-Parp1 (Cell Signalling, Danvers, MA, USA), and mouse anti-Gapdh (Santa Cruz Biotech, Dallas, TX, USA) were used in this study.

### 2.4. Immunofluorescence

For immunofluorescence staining, mESCs at D0 and D8 were immobilized on microscope slides by cytospin, fixed with 3.7% PFA, permeabilized with 0.5% Triton X-100, and blocked with 3% BSA in PBS-Tween 0.05%. Then, they were incubated with primary antibody for 1 h at RT, and after for 1 h in the dark in Alexa-Fluor 488 secondary antibodies. Lastly, cells were incubated with rhodamine-phalloidin 1:1000 to localize filamentous actin and then counterstained with DAPI (4′,6-diamidin-2-phenilindol) 1:100,000 for nucleus visualization.

### 2.5. Statistics

All statistical analyses were performed using a *one-way* ANOVA test. Data are expressed as means ± standard deviation (SD). All experiments were repeated at least three times. A *p* value < 0.05 was considered to be statistically significant.

## 3. Results

### 3.1. Ink4a/Arf Gene Expression Increases in mESCs Induced to Differentiate towards Pancreatic Lineage

**Figure 1:**
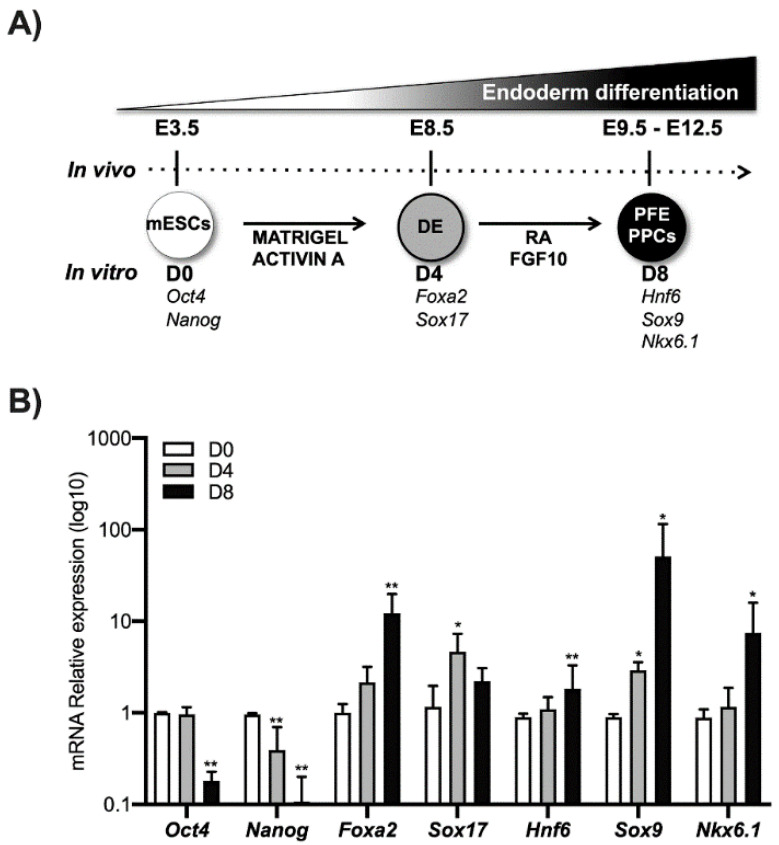
ESCs differentiation toward pancreatic lineage. (**A**) ESCs (D0) were cultured for 4 days treated with activin A (30 ng/mL) (D4), the definitive endoderm (DE) cells were cultured for another 4 days in the presence of both RA and FGF10 to obtain the Posterior Foregut Endoderm (PFE) and enrichment of PPCs (D8). The correspondence of the in vivo and in vitro phases was indicated. (**B**) Pluripotency markers (*Nanog* and *Oct4*), definitive endoderm markers (*Foxa2* and *Sox17*), Posterior Foregut (*Hnf6*), and Pancreatic Progenitor Cell markers (*Sox9* and *Nkx6.1*) were analyzed by qPCR. Data points represent the average of three independent experiments ± SD. * *p* < 0.05; ** *p* < 0.01.

Using an established differentiation protocol [15], E14 mouse ESCs were induced to differentiate into pancreatic progenitors. In particular, mESCs cells expressing *Oct4* and *Nanog* marked as D0 were grown in matrigel and activin A until the expression of the DE markers Foxa2 and Sox17 (typically 4 days, indicated herein as D4). These cells were further treated for 4 days with RA and FGF10 to induce the expression of molecular markers such as such as Hnf6, Sox9, and Nkx6.1 that are typical of the PFE. These cells are highly enriched in PPCs (herein indicated as D8) and represent a good in vitro model to investigate the molecular mechanisms that occur during pancreatic differentiation (Figure 1).

**Figure 2:**
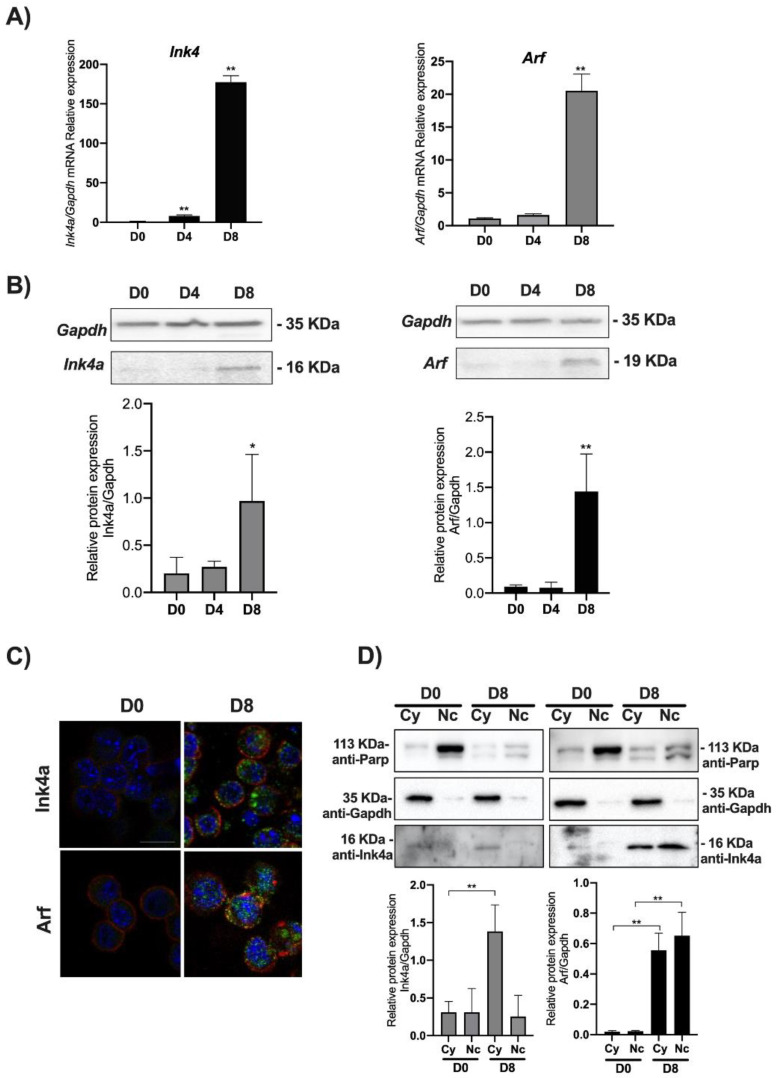
*Ink4a* and *Arf* expression increase in PPCs. ESCs (D0) were induced to differentiate towards PPCs (D4 and D8) and *Ink4a* and *Arf* gene expression analyzed by qPCR, Western blot, and immunofluorescence staining. (**A**) Relative *Ink4a* and *Arf* gene expression vs. Gapdh is reported as folds increase. (**B**) Western blots and densitometric analysis of Ink4a and Arf protein levels relative to Gapdh. (**C**) Merged images of the immunofluorescence staining at D0 and D8 performed with anti-Ink4a and Arf antibodies (*green*), rhodamine-phalloidin (for F-actin, *red*), and DAPI (for DNA, *blue*). Images were taken with a Zeiss confocal laser-scanning microscope LSM 510 (Oberkochen, Germany), scale bar, 15 μm. (**D**) Subcellular fractionation of ESCs at D0 and D8: Western blot analysis of equal amounts of cytoplasmic (30 μg) and nuclear (30 μg) protein extracts. Efficiency of cellular fractionation was checked with anti-Parp (for nuclei) and anti-Gapdh (for cytoplasm) antibodies. Normalized Ink4a and Arf band intensities are expressed relative to Gapdh expression in each experimental point. The data are expressed as average of three independent experiments ± SD, * *p* < 0.05; **, *p* < 0.01.

We thus analyzed the level of expression of the *Ink4a/Arf* locus’ products evaluating both the mRNA and protein levels of *Ink4a* and *Arf* by qPCR and Western blot. A transcriptional analysis of *Ink4a* and *Arf* genes showed that their expression increases as long as the cell’s differentiation proceeds toward the PPC’s lineage. The *Ink4a* gene expression significantly increased at DE (D4) by around 20 times compared to the control (D0), while in PFE (D8), its transcriptional expression was increased by nearly 200 times compared to the control (Figure 2A).

*Arf* gene expression mirrors the kinetics of *Ink4a*. While remaining similar between D4 and D0, the transcript’s level is significantly increased by 20 times in D8. Quantification of Ink4a and Arf immunoblots showed a sharp increase of both proteins in D8 compared to D0 (see graphs in Figure 2B). The immunofluorescence staining confirmed an increased expression of both proteins in D8 (Figure 2C and Figure S2). These results were further confirmed for both proteins by nuclear/cytoplasmic fractionation in D0 and D8 (Figure 2D). Ink4a appeared poorly expressed in D0, acquiring a clear cytoplasmic localization in D8 (Figure S1). On the other end, Arf showed a sharp increase both in the nucleus and cytoplasm in D8 (Figure 2D and Figure S3).

### 3.2. Ink4a/Arf Gene Expression Correlates with Endoderm-Nepn Cells

**Figure 3:**
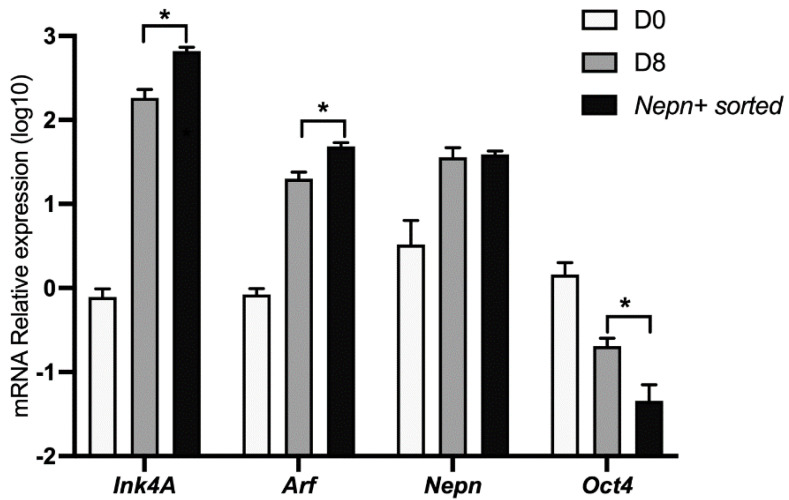
*Ink4a* and *Arf* expression marks Nepn-expressing cells. mESCs were induced to differentiate for 8 days as described, and cells expressing Nepn-expressing cells were isolated by FACS. Total RNA was extracted and analyzed by qPCR to check *Ink4a*, *Arf*, *Nepn,* and *Oct4* gene expression. Data points represent the average of independent experiments performed in triplicate ± SD. *, *p* < 0.05.

It has been shown that the *Nepn* gene marks the early stages of murine endoderm specification and is required for correct endoderm differentiation [14,15]. To further characterize the kinetics of the *Ink4a* and *Arf* expression in our cellular system, we differentiated ES cells as described before and analyzed the locus’ expression in a pure subpopulation of *Nepn* expressing cells. Differentiating cells were sorted by FACS in order to obtain a population enriched in *Nepn* expressing progenitor cells. When we analyzed *Cdkn2a*’s transcript levels by qPCR, we found a significant enrichment of both *Ink4a* and *Arf* expression in Nepn-sorted cells. Moreover, a comparison of the expression level dynamics strongly suggests that the locus expression is confined in the Nepn+ cell state (Figure 3).

### 3.3. DNA Methylation and Chromatin Acetylation Affect Ink4a and Arf Gene Expression in ESCs

**Figure 4:**
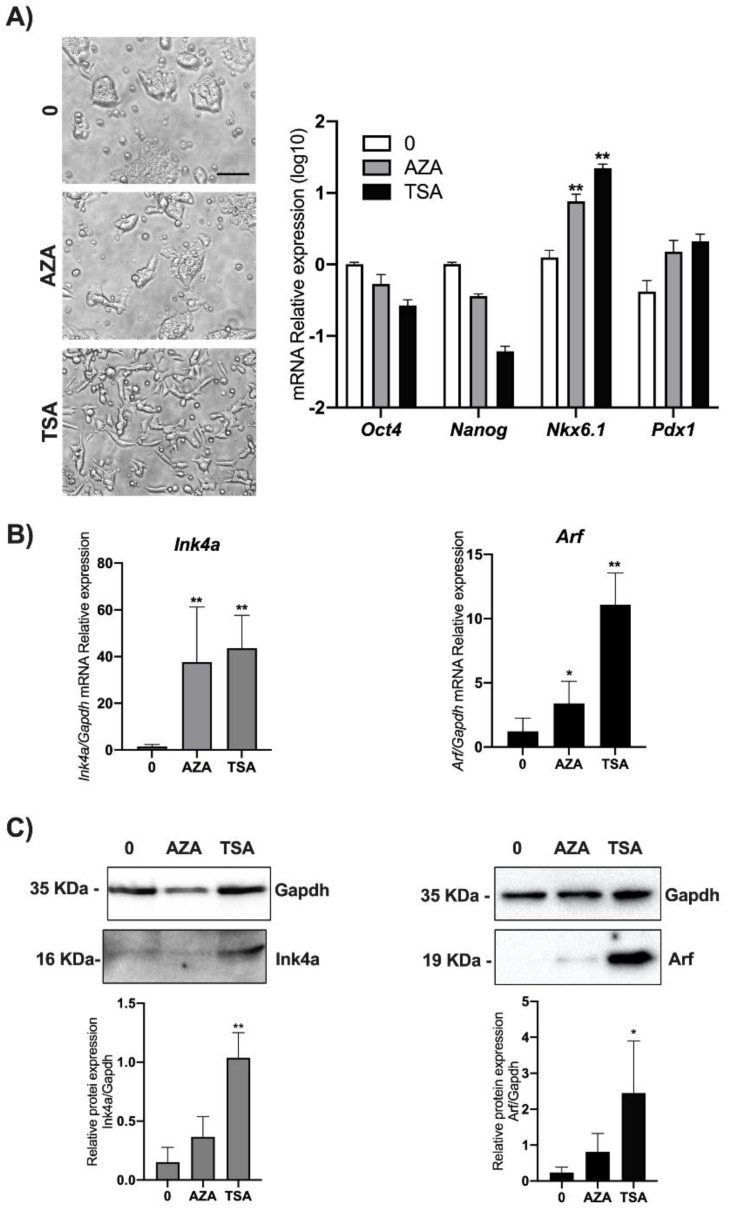
Effect of AZA and TSA on *Ink4A* and *Arf* gene expression in mESCs. ESCs were treated either with 25 μM AZA or 100 nM TSA and with 0.1% DMSO as control (0). (**A**) After 24 h, treated and control mESCs were imaged using phase-contrast microscopy with a 10× objective (left) (scale bar, 60 μm). The experiment was performed in triplicate, and different fields were observed. Total RNA and proteins were isolated for each experimental point, and the levels of genes expression of *Oct4*, *Nanog*, *Nkx6.1,* and *Pdx1* were analyzed as experimental control (right). Ink4a and Arf expression was analyzed by (**B**) qPCR and (**C**) Western blot assays. Data are shown as mean ± SD from three independent experiments, normalized with Gapdh, and reported with respect to the control (0). *, *p* < 0.05; **, *p* < 0.01.

It is known that the *Cdkn2a* locus is not expressed in ES and in iPS (induced Pluripotent Stem) cells but acquires the epigenetic marks of a bivalent chromatin domain, thus retaining the ability to be reactivated if required after differentiation onset [19,20]. Epigenetic suppression of the INK4A-ARF locus has been widely shown, and greatly promotes survival and cloning efficiency in individualized human ESCs via deposition of the H3K27me3-mark to INK4A-ARF locus [21,22]. In order to verify if epigenetic modifications, specifically DNA methylation and chromatin deacetylation, were responsible for the Ink4a and Arf regulation in ESCs, we performed treatments of cells with widely used epidrugs. In particular, we used either the demethylating agent AZA or the HDAC inhibitor TSA. mESCs were treated with 25 µM AZA or with 100 nM TSA. Twenty-four hours post-treatments, *Ink4a* and *Arf* expression in mESCs cells was simultaneously analyzed by qPCR and Western blot. Before lysis, cells were observed by phase-contrast microscopy, and we noticed that cells treated with TSA, in contrast to AZA treated cells, acquired an elongated morphology (Figure 4A left). The analysis of pluripotency markers such as *Oct4* and *Nanog* [23,24] and differentiation markers such as *Nkx6.1* and *Pdx1* [25] are in line with the epigenetic pattern of regulation of these genes suggesting the priming of a differentiation program (Figure 4A right).

While we found that both treatments significantly increase both Ink4a and Arf transcriptional levels, analysis by Western blot revealed a significant increase only upon TSA treatment (Figure 4B,C and Figure S4).

## 4. Discussion

In this study, by using mouse embryonic stem cells induced to differentiate as a model system, we analyzed the expression of the *Cdkn2a*/*Arf* locus during pancreatic lineage commitment. Western blot and immunofluorescence analysis confirmed an increase in both protein products. Interestingly, we found that *Cdkn2a* locus activation mainly takes place in cells expressing *Nepn*, a factor that marks the genesis of pancreas bud being expressed in a naïve pancreatic cellular state resembling PPC-like specification. To further confirm our data, we used data obtained by mRNA expression profiles of hPSCs differentiating along the pancreatic beta-cell linage [26]. Using repository data from GEO (http://www.ncbi.nlm.nih.gov/geo (accessed on 1 January 2021)) (GSE42094), we performed a bioinformatic analysis looking for both ARF and INK4a expression. This analysis showed the activation of the locus, in agreement with our results in mice, although with different kinetics (data not shown).

Physiological expression of ARF during development has been described in a precise time frame during the differentiation of several tissue types, such as the developing eye, spermatogonia [27], differentiating keratinocytes [28], epithelial derivative, and the yolk sac [29]. The transcriptional activation of these two tumor suppressors has been related to their function of monitoring and restricting aberrant mitogenic signals arising during cell differentiation. Several studies highlighted the importance of proper heterochromatin establishment to promote cell differentiation and the maintenance of cell identity during embryogenesis. Epigenetic regulation of chromatin structure has a key role in the control of cell differentiation and cell identity in embryogenesis. The *INK4-ARF* locus is epigenetically silenced thanks to the function of the Polycomb group of proteins [19,21] Importantly, repression of the *Ink4a/ARF* locus represents a roadblock during iPS (induced Pluripotent Stem Cells) generation [20,29], in line with the role of epigenetic regulation of this locus in ES cells homeostasis. While p16Ink4a and p19Arf induction in cultured primary cells cause senescence [30], Bmi-1 overexpression can prevent senescence and extend their replicative lifespan by reducing p16Ink4a and p19Arf expression [31]. Our results are in line with the epigenetic regulation of the locus. We observed that the treatment of undifferentiated cells with epigenetic drugs such as AZA and TSA, in addition to inducing some features of endoderm’s differentiation, also induces the expression of both p16Ink4a and p19Arf, thus suggesting that an epigenetic remodeling of the locus could take place during pancreatic differentiation, in line with studies in other cellular systems [32]. Interestingly, ARF expression upon TSA treatment is characterized by the acquisition of an elongated cellular morphology. This observation, together with previously published data about ARF’s role in cytoskeleton remodeling [18], suggests that ARF expression in pancreatic progenitors could have a role in this phenomenon. Besides, Ink4a and Arf ectopic expression correlates with a NeuroD1 increase, mainly involved in beta cells differentiation of pancreatic development [33], and p16Ink4a activation has been reported in human pluripotent stem cells (hPSCs) induced to differentiate into cardiomyocytes [34]. The *CDKN2A* locus has been shown to affect cell metabolism by influencing glucose homeostasis, β-cell functions and mass, hepatic gluconeogenesis, and lipid storage [35]. Genome-wide association studies (GWASs) have unequivocally linked the CDKN2A locus with human diabetes risk, although the mechanisms remain uncertain [21,36,37,38,39]. Moreover, it is known that CDKN2A mutation occurs in early PDAC (pancreatic ductal adenocarcinoma) [40,41] and, CDKN2A promoter hypermethylation is related to malignant tumor development [42].

Our data are in agreement with those found in human ES during endodermal-pancreatic differentiation, prompting us to think that the expression of Ink4a and Arf in PPC is due either to an organ regeneration mechanism or to a stress response. Given that alteration in PPCs homeostasis, besides being fundamental to physiological processes such as organ development, are also a key step in pathological events such as cancer, elucidation of the mechanisms governing tissues development is critical to understand the cause of organ disorders and essential for the development of new therapies focused on tissue and organ regeneration.

## Figures and Tables

**Table 1:** List of genes and sequences of primers used for relative gene expression analysis by qPCR.

Gene	Name	Primer fw 5′–3′	Primer rv 5′–3′
*Foxa2*	Forkhead Box A2	CTGGGAGCCGTGAAGATGGAAG	TCCAGCGCCCACATAGGATG
*Gapdh*	Glyceraldehyde 3-phosphate dehydrogenase	AATGGTGAAGGTCGGTGTG	GAAGATGGTGATGGGCTTCC
*Hnf6*	Hepatocyte nuclear factor 6	CAAAGAGGTGGCGCAGCGTATC	GCTCTTTCCGTTTGCAGGCTG
*Ink4a*	p16Ink4a	CCCAACGCCCCGAACT	GCAGAAGAGCTGCTACGTGAA
*Nanog*	Nanog homeobox	AACCAGTGGTTGAAGACTAGCAATGGTC	TTCCAGATGCGTTCACCAGATAGC
*Nepn*	Nephrocan	AACCTCTGTGTTGGACAATGC	TCAGAGTTTTGAAGGTGTCATTTT
*Nkx6.1*	Nk6 homeobox1	ACTTGGCAGGACCAGAGAGA	AGAGTTCGGGTCCAGAGGTT
*Oct4*	Octamer-binding transcription factor4	CCGTGTGAGGTGGAGTCTGGAGAC	CGCCGGTTACAGAACCATACTCG
*Pdx1*	Pancreatic and duodenal homeobox 1	GCTCACCTCCACCGGACCTTC	GGGTCCTCTTGTTTTCCTCGGG
*Arf*	p19Arf	TGAGGCTAGAGAGGATCTT	CGTGAACGTTGCCCATCAT
*Sox9*	Sry-Box transcription factor 9	GGTCTGCCTGGACTGTATGTGGATG	CTGTCCGATGTCTCTCTGCAGGAG
*Sox17*	Sry-Box transcription factor 17	GCCGATGAACGCCTTTATGGTG	CATGCGCTTCACCTGCTTGC

## Data Availability

Not applicable.

## Supplementary Materials

The following are available online at https://www.mdpi.com/article/10.3390/biom11081124/s1, Figure S1: Full-length Western blots images of cropped immunoblots of Figure 2B, Figure S2: Immunofluorescence assays for Ink4 and Arf subcellular localization of Figure 2C, Figure S3: Full-length Western blots images of cropped immunoblots of Figure 2D, Figure S4: Full-length Western blots images of cropped immunoblots of Figure 4C.
