# Supplementary material for: Pancreatic Progenitor Commitment Is Marked by an Increase in Ink4a/Arf Expression
Source: Biomolecules. 2021 Jul 30;11(8):1124. doi: 10.3390/biom11081124 (PMC8392192; doi:10.3390/biom11081124)

SUPPLEMENTARY INFORMATIONS

Pancreatic progenitor commitment is marked by an increase in *Ink4a/Arf* expression

Figure S1. Full-length western blots images of cropped immunoblots in Figure 2B. Blue dashed squares correspond to the bands showed in the ultimate panel in the manuscript.

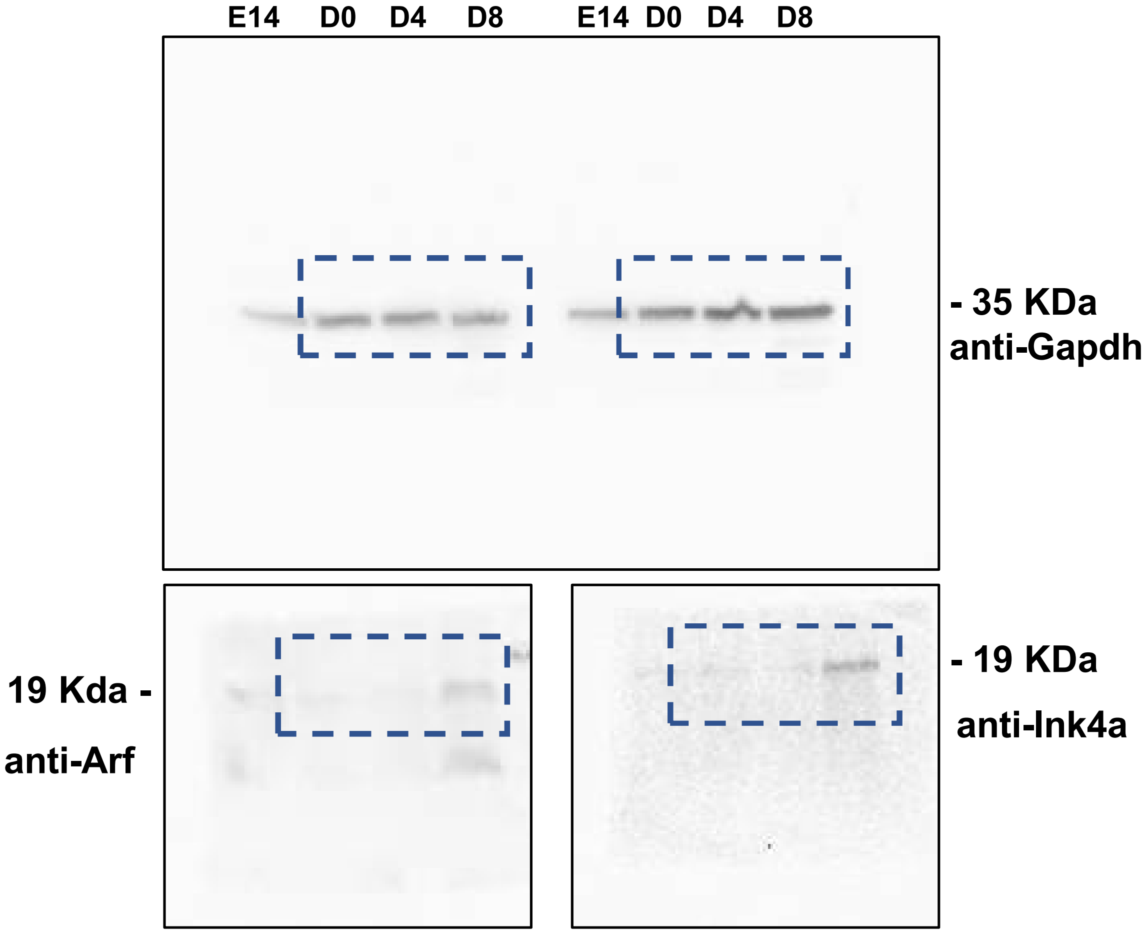

Figure S2. Immunofluorescence assays for Ink4 and Arf subcellular localization. ESCs (D0) were induced to differentiate into PPCs (D8) and subjected to immunofluorescence with Ink4a and Arf antibodies (*green*), actin (*red*) for cytoplasm and DAPI (*blue*) for nucleus staining. The merge images were cropped (as in Figure 2C) and stained using Fiji (ImageJ) program.

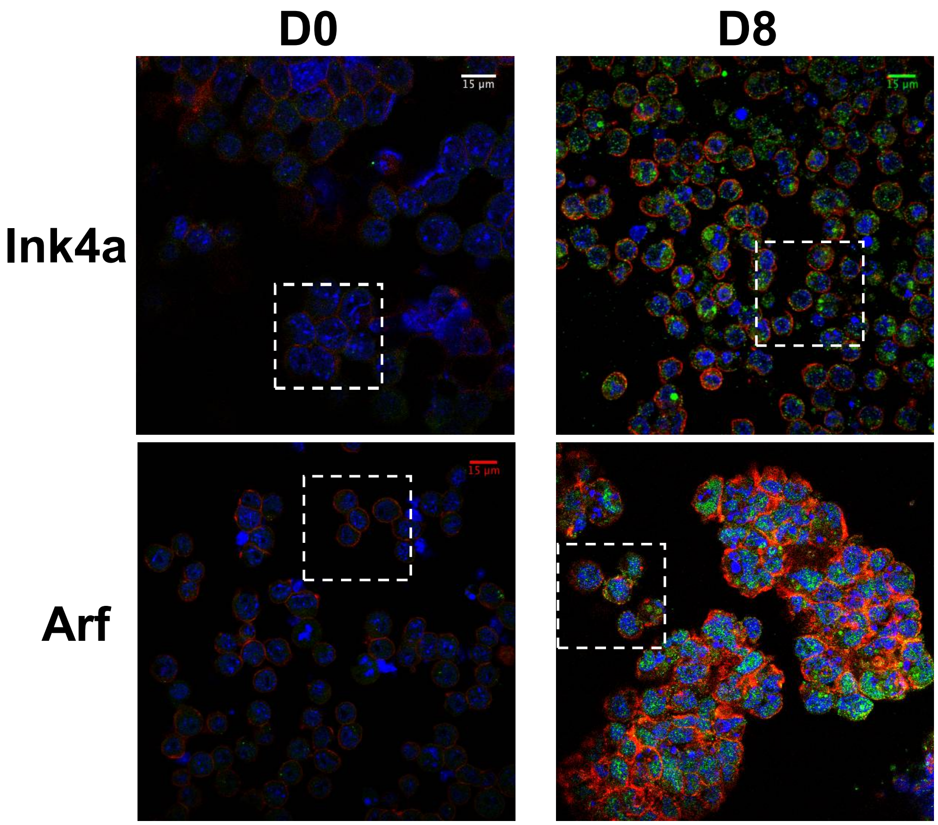

Figure S3. Ink4A and Arf protein differentially expressed in PPCs. Full-length western blots images with cropped in Figure 2D, anti-Ink4a and anti-Arf antibodies were used for our protein staining, anti-Parp was used as nuclear and anti-Gapdh as cytoplasmic markers, respectively.

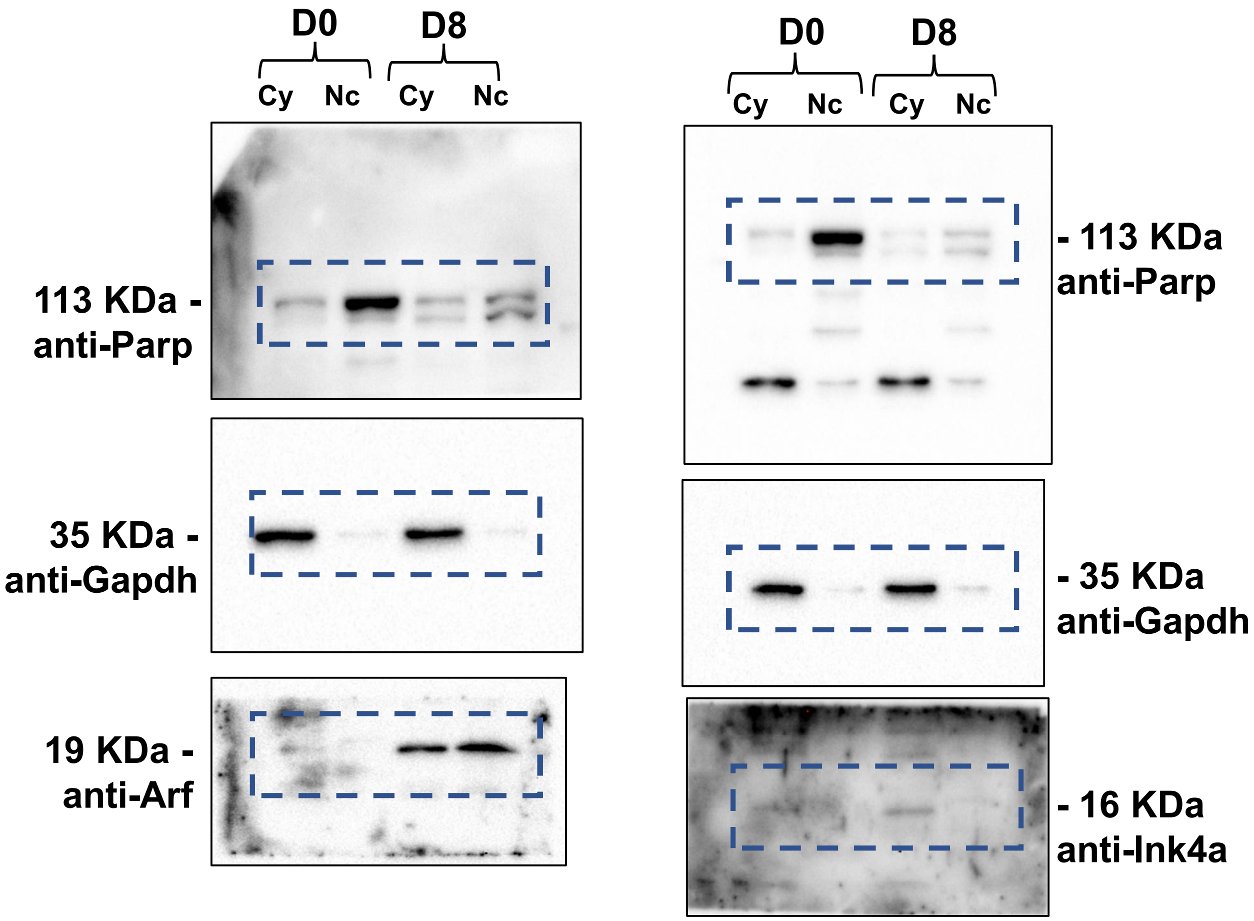

Figure S4. Full-length western blots images of cropped immunoblots of Figure 4C. Blue dashed squares correspond to the bands showed in the ultimate panel in the manuscript. DMSO: negative control.

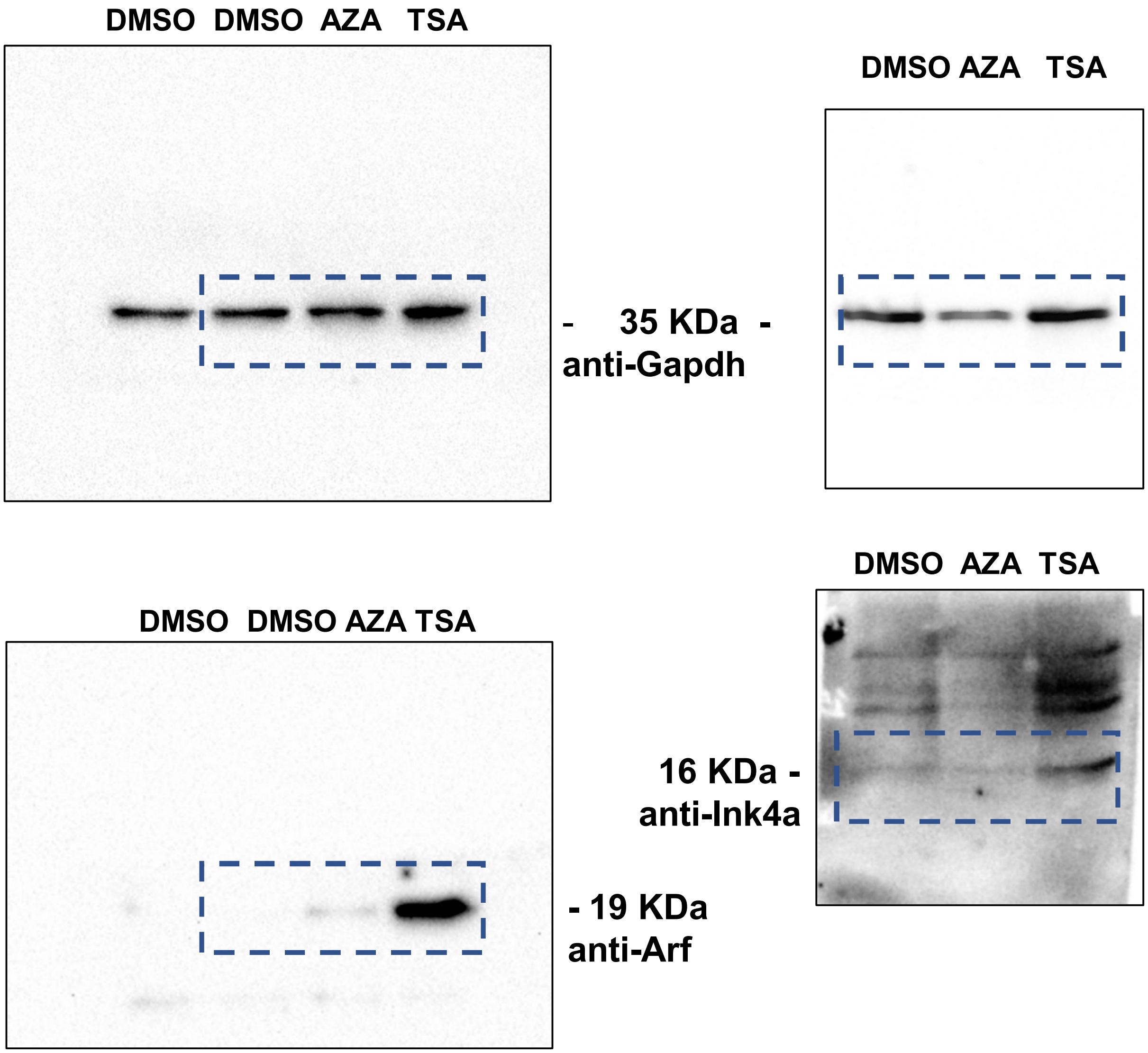

Supplement: Supplementary file 1 [file biomolecules-11-01124-s001.zip › biomolecules-1161528-supplementary.pdf]
